# Long-term survey of a syringe-dispensing machine needle exchange program: answering public concerns

**DOI:** 10.1186/1477-7517-11-16

**Published:** 2014-05-22

**Authors:** Catherine Duplessy, Emmanuel G Reynaud

**Affiliations:** 1 SAFE, 11 avenue de la Porte de la Plaine, Paris 75015, France; 2 School of Biology and Environmental Sciences, University College Dublin, Belfield, Dublin 4, Ireland

## Abstract

**Background:**

Syringe-dispensing machines (SDM) provide syringes at any time even to hard-to-reach injecting drug users (IDUs). They represent an important harm reduction strategy in large populated urban areas such as Paris. We analyzed the performance of one of the world's largest SDM schemes based in Paris over 12 years to understand its efficiency and its limitations, to answer public and stakeholder concerns and optimize its outputs.

**Methods:**

Parisian syringe dispensing and exchange machines were monitored as well as their sharp disposals and associated bins over a 12-year period. Moreover, mechanical counting devices were installed on specific syringe-dispensing/exchange machines to record the characteristics of the exchange process.

**Results:**

Distribution and needle exchange have risen steadily by 202% for the distribution and 2,000% for syringe recovery even without a coin counterpart. However, 2 machines out of 34 generate 50% of the total activity of the scheme. It takes 14 s for an IDU to collect a syringe, while the average user takes 3.76 syringes per session 20 min apart. Interestingly, collection time stops early in the evening (19 h) for the entire night.

**Conclusions:**

SDMs had an increasing distribution role during daytime as part of the harm reduction strategy in Paris with efficient recycling capacities of used syringes and a limited number of kits collected by IDU. Using counting devices to monitor Syringe Exchange Programs (SEPs) is a very helpful tool to optimize use and answer public and stakeholder concerns.

## Background

The primary public health strategy to prevent the transmission of blood-borne viruses such as HIV among and from people who inject drugs has been to provide extensive and free access to sterile needles and syringes. Following the HIV epidemic among drug users in the USA, several attempts to reduce needle and syringe sharing were initiated to reduce the spread of the virus. Among those, Syringe Exchange Programs (SEPs) became a mainstream tool of harm reduction programs around the world. Their benefits have been clearly established for infectious risks [1]. Many types of SEPs have been developed, ranging from pharmacies, locally run outlets, mobile units (vans) and syringe vending machines (SVM) or syringe-dispensing machines (SDM) [2,3].

Since the mid 1980s, harm-reduction strategies have been criticized by local communities and stakeholders questioning their effectiveness and their role in promoting drug use. One way to address those critics has been to measure their public health efficacy by following virus prevalence among drug users and general population, before and after SEP opening or by comparing sectors with or without SEPs [4]. But despite positive results, hostile reactions from local communities are still common, and a new term as been forged to define such opposition: ‘NIMBYism’ (‘not in my back yard’) [5]. They argue that SEPs may encourage drug consumption, develop drug traffic, create social disorder and increase public insalubrities. But the critics vary greatly from one neighbourhood to the next, depending on social context, ethnicity and unemployment rate, for example. Secondly, elected representatives are questioning SEP efficiency as they invest public money in those schemes and related organizations and structures. They are important supports for SEPs as long as they can prove their public health efficacy or answers public concerns with well-structured reports and surveys including long-term monitoring and calculation of cost-efficiency parameters (IDU cost per year, dirty syringe recycling rate, etc.).

Similar to soda vending machines, SDMs (or SVMs) can deliver sterile syringes and related paraphernalia (cups, swabs, etc.) 24 h a day and 7 days a week all year long without supervision. They do so against money, used syringes or freely distributed coins. Those machines regularly attracted a segment of the IDU population that are not reached via SEPs or pharmacy sales [6] and a broader range of injectors [7]. SDMs are regarded as a cost-efficient solution to deliver syringes at any time of the day to any type of user in comparison to pharmacies or social workers usually submitted to time constraints. However, there is a limited amount of literature on their effectiveness mainly based on questionnaires [8]. SDMs have been introduced in over a hundred European and Australian cities [9,10]. One such harm-reduction scheme based on SDM has been run in Paris for over a decade with 34 units by 2014. This extensive network of units spread over a large city with diverse neighbourhoods has attracted extensive criticisms from local communities, stakeholders and elected representatives.

We analyze quantitatively the dynamic of an SEP using SDMs over 12 years. In order to define precisely the characteristics of the SDMs, we have developed counting devices that allow us to time and quantify the exchange process. This permits us to draw general conclusions about the dynamic and effectiveness of the SEP to answer critics and concerns in addition to improving the SEP performance to better answer users' needs.

## Methods

### Syringe-dispensing machines

Three types of syringe dispensing machines were monitored: distributing machines that provided a prevention kit (named Kit^+^®, EDEC Laboratories, Cournon, France) against a coin, exchanging machines that provide a coin against a used syringe and collecting machines that do not deliver any counterpart. The machines are of the following brands: AVAL (Issy-les-Moulineaux, France), MGR (Chaux, France), Vibromat (Noyelles-les-Vermelles, France) and Sielaff (Collégien, France). All SDMs provide Kits^+^® (EDEC Laboratories). Each Kit^+^® contains two 1-ml syringes with a fused 0.33 × 12.7-mm gauge needle, two 5-ml water flasks, two alcohol swabs, two Stéricups® (a rigid plastic container that contains one cup, one filter and one dry swab) and one condom. Containers (EDEC Laboratories and EURECA Society, Langon, France) used for syringe collection are specialized infection trash collection containers of 5-, 25- or 50-l capacity in exchanging machine and 50-l containers for syringe and trash collection in collecting machines. The monitoring of the syringe containers as well as the associated bins was performed as follows. Each object was extracted with tweezers, counted and classified by two different persons.

### Counting devices

In order to monitor the syringe exchange process within SDMs, the counting devices were specifically designed for this project (IMPACT GmbH, Cologne, Germany). They were optimized for Distribox and Changebox models. Those devices were installed alternatively on particular SDMs of interest. They record the time of every operation of the SDM by monitoring the movement of the SDM drawer block: used syringe insertion, coin collection, coin insertion and prevention kit collection. All data are recorded on a flash card. The data collection is done at regular interval via a card reader and processed using numerical analysis software.

### Data analysis

Kits and syringe distribution in SDMs were routinely recorded by SAFE staff during their daily or weekly distribution route using established reporting forms. All forms were transcribed in an Excel spreadsheet, saved, stored and backed up on a local server. Counting device data were recorded using a card reader. The data was saved as a text file containing date, time and unit code. Those were converted into Excel spreadsheets, saved, stored and backed up. The analysis was performed using Excel analysis tools to obtain descriptive statistics, including mean and standard deviation (SD). Finally, user interaction data were recorded by SAFE social workers team on site on specifically design forms then recorded electronically at the office.

## Results

### History of the SAFE Parisian dispensing machine SEP

**Figure 1:**
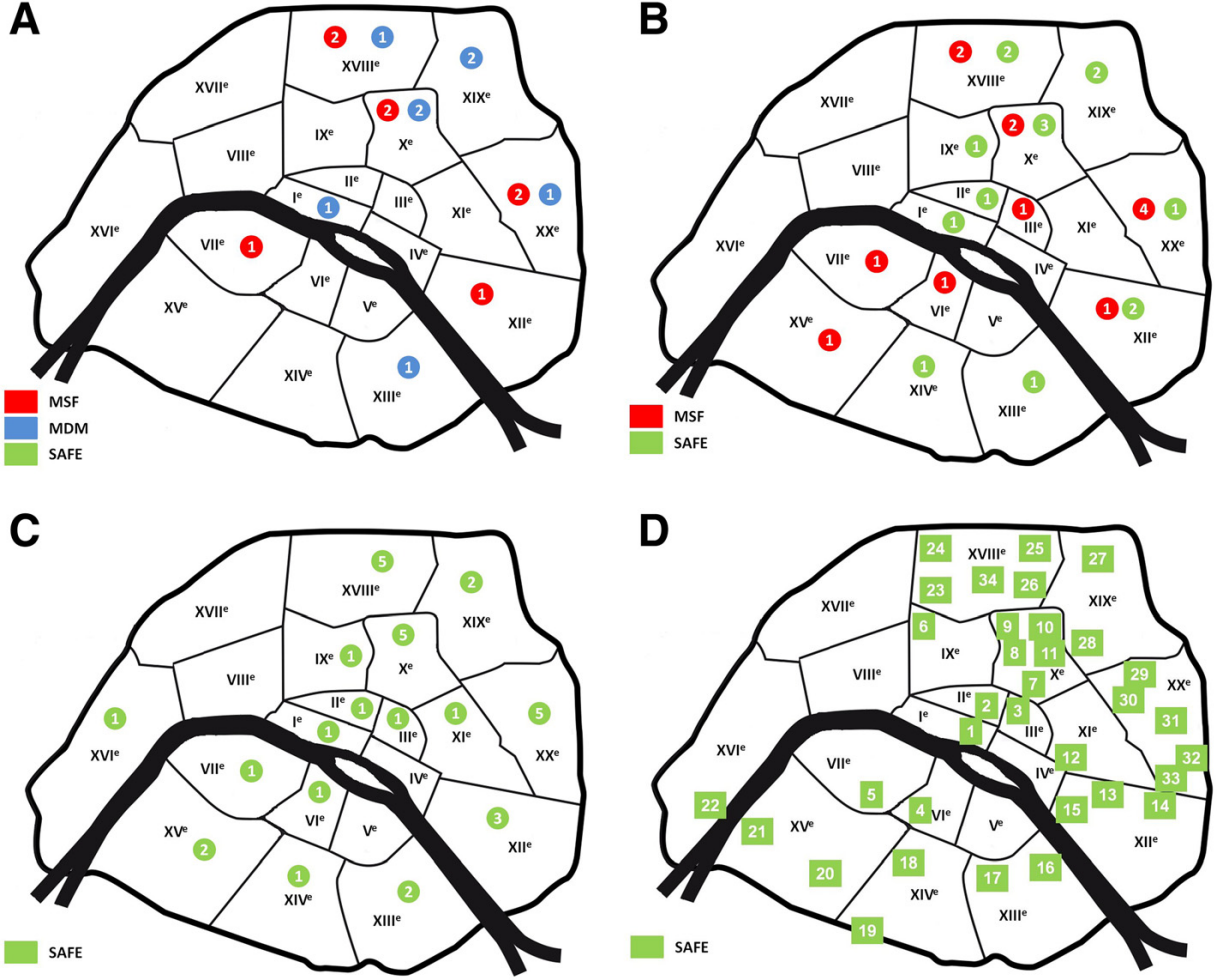
**The SAFE dispensing machine SEP. (A)** The numbers of implementations in 2000. **(B)** The status of the SEP in 2004. **(C)** Actual SAFE SEP by dispensing machines. **(D)** Activity of the SAFE SEP in 2009.

We initiated our analysis by studying the historical processes that led to the establishment of this SEP and its evolution over a 12-year period under different organizations and management with extensive material changes. In Paris, the dispensing machine SEP was initiated during the 1990s by two humanitarian organizations: ‘Médecins Sans Frontières (MSF)’ and ‘Médecins du Monde (MDM)’. Each one developed a specific strategy. MDM implemented dispensing machines within the drug consumption zones, coupling distributors and exchangers. MSF organized a partnership with pharmacists to install only distributors in front of their shops, to encourage the IDUs to enter the pharmacy and to be in contact with health professionals (Figure 1A,B). In 2000, MDM stopped their action, followed by MSF in 2004, and they both donated their dispensing machines to SAFE. This association decided to develop the program where access to syringes was still difficult or insufficient, especially near railway stations. In partnership with French authorities (state, region and town representatives), SAFE has evaluated all the sites to define problems, inadequacies and technical difficulties. First, dispensing machines distributing less than 700 kits per year were moved to sites where they were expected to perform better. Secondly, SAFE improved the existing machines by replacing the coupling exchanger-distributor for a sturdier version, adding exchangers to distributors (MSF implementations) and moving some machines to better new location sites. Finally, the association further developed the implementation of dispensing machines by establishing new sites for a better coverage of the capital city and its different quarters (Figure 1C).

Within a few years, SAFE has improved greatly the spatial coverage by implementing new machines in depleted zones and sub-optimized regions or reorganizing the machine distribution to reduce the distance between users and the service provided (Figure 1C). This approach allows easier access to harm reduction equipment even in less favored districts where users are hard to reach and less inclined to interact with dedicated harm reduction programs. However, the major part of its activity (70%) is found on the North West quarter of Paris city. One specific district of the Paris city centre known as the 10th arrondissement, where two major train stations are located (Gare du Nord and Gare de l'Est), represents more than 50% of the total activity, but overall, the southern city centre activity (districts 6th, 7th, 12th, 13th, 14th, 15th and 16th) has risen and more than doubled over the 2004 to 2012 period from 9,500 kits to 19,500 distributed and today represents 14% of the SEP overall (Figure 1D).

The Paris-based SEP has evolved over 12 years with nearly constant monitoring and represents a perfect set-up to analyze long-term evolution over time of an SDM-based SEP. Interestingly, the original dispensing machines were replaced and moved to new locations, promoting the coupling of exchanger with distributor.

### Performances of the SAFE SEP

The SAFE SEP has been monitored over a 12-year period (1999 to 2012). The data were acquired from the two original actors: MDM (1999–2000) and MSF (1999–2004) as well as the monthly follow-ups performed by SAFE staff and social workers since 2000 to 2004. Due to the large modification and reorganization of the implementations, we have divided our study in two parts. Firstly, we have followed up the overall performance of the entire SEP over time. On the other hand, we have analyzed specifically a number of sites that have been unchanged over time.

### General results

**Figure 2:**
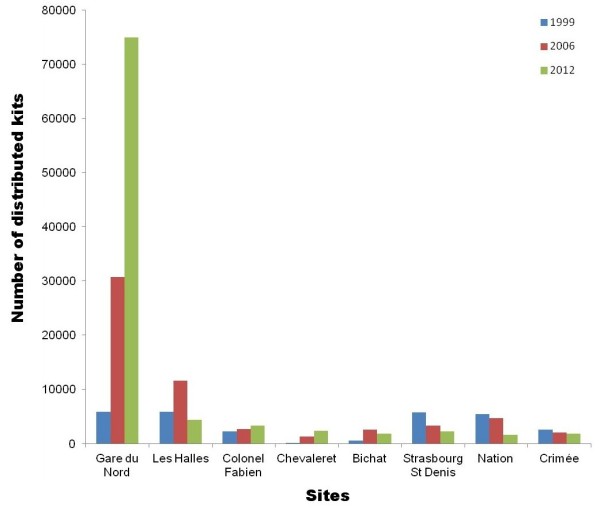
**Site-to-site variation over time.** Eight sites were followed for their distribution activity over 10 years (blue: 1999, red: 2006 and green: 2012).

Distribution and needle exchange have been rising steadily until 2007, from 51,776 to 156,451 Kits© (103,552 and 312,902 syringes, respectively) and 11,719 to 250,300 syringes (88,151 exchanged for a token and 162,149 unrequited) (Figure 2). This represents an increase of 202% in distribution over 12 years (20%/year) and an increase of 2,000% recovery (up 650% for strict exchange). The whole activity of the SAFE SEP decreased in 2008, to rise again in 2009 up to now. In 2012, the increase for distribution is 10% over 2011. However, site-specific activity is very variable, both in terms of distribution and rate of recovery. A few sites are the exception and keep an almost constant activity, always important (Gare du Nord, 21%; Barbès, 20%; Les Halles, 8%) or slightly less important but steady (Sebastopol, 5%; Place d'Italie, 1.5%).

### Performance over time on specific sites

**Figure 3:**
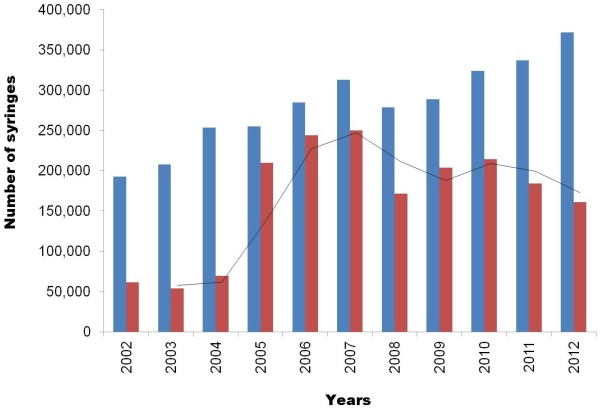
**Syringe distribution over time.** Blue: syringe distribution, red: syringe recycled and black line: global syringe collection.

As mentioned above, the numbers of sites that have remained unchanged over time is limited. However, we have isolated eight sites that we could follow over 10 years: Gare du Nord, Les Halles, Colonel Fabien, Chevaleret, Bichat, Strasbourg Saint Denis, Nation and Crimée (Figure 3). As shown previously, there has been a steady and significant increase of kit distribution in the SAFE SEP; however, this is not an overall increase. Gare du Nord and Chevaleret show a correlated increase, but not the other six sites. This pointed out a more complex behaviour of the IDU population that required a more precise description of the SEP at the site level rather than the overall SEP.

### Annual variations of syringe distribution

**Figure 4:**
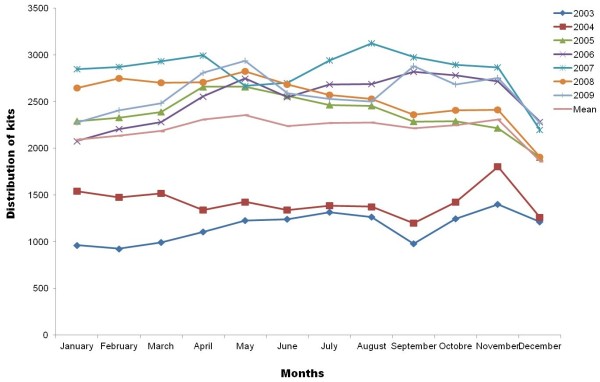
**Annual variation of the SAFE SEP over time.** Due to limited monthly follow-up prior to 2002, the SAFE SEP was monitored for 6 years only.

We took advantages of the monthly follow-up introduced by SAFE up to 2009 to further analyze the SEP characteristics. The distribution is almost constant (Table 1 and Figure 4). However, three slight variations can be observed. The lowest activity level occurred in December and January, while the highest activity level was in April and May. Those peaks can be explained by technical as well as social parameters (see the ‘Discussion’ section below). It is also interesting to notice that the 2 months of July and August are also among the highest months for distribution and the operation of the SAFE team especially at the Gare du Nord machine. This is essentially correlated with the closing down of a nearby SEP over summer.

**Table 1:** Annual variation of the SAFE SEP over time

**Year**	**January**	**February**	**March**	**April**	**May**	**June**	**July**	**August**	**September**	**October**	**November**	**December**	**MEAN**
2003	961.78	924.50	991.33	1,105.56	1,226.44	1,239.50	1,315.56	1,263.33	977.50	1,245.78	1,400.50	1,212.73	1,155.38
2004	1,542.00	1,473.25	1,516.00	1,340.22	1,425.78	1,337.75	1,384.89	1,374.00	1,198.75	1,423.33	1,803.75	1,259.64	1,423.28
2005	2,290.44	2,328.50	2,388.00	2,658.00	2,658.22	2,563.00	2,462.89	2,452.89	2,285.75	2,291.33	2,214.50	1,902.91	2,374.70
2006	2,077.56	2,205.50	2,282.44	2,555.33	2,747.33	2,549.25	2,684.22	2,690.67	2,820.00	2,782.44	2,718.50	2,283.09	2,533.03
2007	2,848.00	2,872.25	2,932.67	2,995.78	2,668.00	2,700.75	2,942.89	3,124.67	2,977.25	2,894.44	2,865.25	2,196.91	2,834.91
2008	2,645.56	2,750.00	2,702.89	2,706.00	2,823.78	2,685.25	2,570.44	2,530.00	2,361.50	2,408.67	2,411.50	1,904.91	2,541.71
2009	2,276.22	2,407.00	2,484.00	2,808.22	2,938.44	2,590.00	2,530.00	2,502.67	2,881.50	2,683.33	2,756.00	2,270.55	2,593.99
2010	2,312.00	2,373.00	2,518.00	2,834.00	2,984.00	2,590.00	3,135.00	2,513.00	2,881.00	1,687.00	2,761.00	2,766.00	2,612.83
2011	3,096.00	2,865.00	3,170.00	3,231.00	3,471.00	3,350.00	3,399.00	3,080.00	3,178.00	3,391.00	3,318.00	3,275.00	3,235.33
2012	3,212.00	3,163.00	3,249.00	3,444.00	3,758.00	3,766.00	3,888.00	3,651.00	3,726.00	3,483.00	3,584.00	3,742.00	**3,555.50**
Mean	*2,326.16*	*2,336.20*	*2,423.43*	*2,567.81*	**2,670.10**	*2,537.15*	**2,631.29**	*2,518.22*	*2,528.73*	*2,429.03*	*2,583.30*	*2,281.37*	2,486.07

Italicized values: lowest months, values in bold: highest months.

### SDM set-up management issues

**Figure 5:**
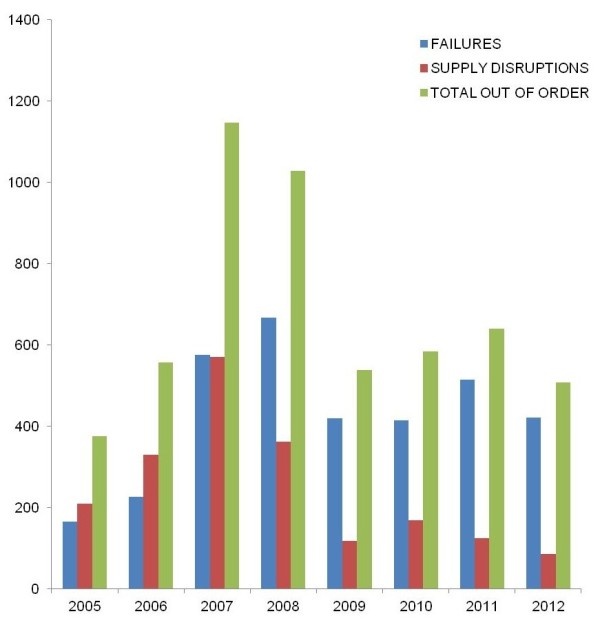
**Evolution of the failures and repairs of the SAFE SEP SDMs.** Eight sites were followed for their distribution activity over 5 years (blue: failures, red: disruptions and green: out of order).

The dispensing machines can be used 24 h a day and 7 days a week but need to be refilled and looked after. The follow-up of failures, repairs and refills is an important measure of the overall activity of such a large set-up. This requires the accurate recording of those events. We have been only able to obtain the records up to 2005 (5 years). The follow-up is divided in two lines of records: failures/repairs and supply disruptions. The latter measure represents a lack of availability (SEP related), while the former can reflect the machine mismanagement, age or vandalism (technical related). There was a significant increase in decommissioned machines from 2005 to 2007, both because of supply disruptions and technical difficulties (Figure 5). This is not simply related to the entire set-up development, since the data weighted to the number of devices or sites are increasing. It is firstly an increase in the number of failures: they related primarily to the aging machinery; second, to the overuse of some of them (29% of failures relate only to the Gare du Nord site in 2007) and finally to the poor quality of one brand. In parallel, we recorded an important increase in the number of supply disruptions; they have more than doubled between 2005 and 2007, despite an increase in refilling tours per day and the establishment of an everyday refilling tour. In particular, the area of Gare du Nord suffers alone 88% of supply disruptions.

### Monitoring a SDM SEP

To better understand the site-by-site variation, we have equipped distributors and exchangers with automatic counting devices to measure the distribution flux over time. This non-invasive strategy (invisible by the IDUs) was designed to define the speed of the processes as well as their extent during the day or in between distribution or exchange actions. This allows a non-biased measure of the number of kits per user and their behaviour qualitatively and quantitavely. Additionally, this can be used to define the distribution window per day to understand supply disruption.

We followed two sites: Barbès and Gare du Nord for the distribution and recycling activities. This first pilot study was primarily intended for syringe collection (517 events). The syringe collection time in a syringe-distributing machine is 14 s (coin against a new kit) and 18 s in the case of an SDM coupled with an exchanging unit (dirty syringe against a coin then for a new kit). They are very similar values. The additional 4-s difference (+28%) could be a simple effect of the number of steps required (opening the drawer, syringe introduction, taking the token and then collecting the kit). The time in between two IDU syringe collection is also similar (19 min and 45 s for SDM and 23 min and 30 s for SDM + exchanging unit (+18%)), but is relatively longer when considering the collection time (84 and 78 s longer, respectively). The number of kits taken per IDU is 1.88 (3.76 syringes), and the number of recycled syringe per user is 2.3 syringes. The recycling rate is 61%, while the expected rate is 50% (one recycled syringe for one token provides one kit containing two syringes).

The follow-up of the overall activity in Gare du Nord and Barbès pointed out an early ending of its activity (syringe recycling: 21 h/21 h 30 min; collection: 19 h/20 h 45 min) and a empty time slot of 15 h 30 min and 15 h 15 min, respectively. The use of counting devices on SDMs is a very useful tool that allows the follow-up of the distribution and recycling processes with limited biases. This is very efficient for defining limitations, understanding problems and solving them.

### Answering public concerns

Our approach using large data collection on an entire SDM scheme over time using counting devices has been well received by the different players involved in harm reduction in France (national drug initiatives, local authorities, etc.). SAFE has been requested to provide reports to national initiatives, administrative authorities and organizations (Table 2). SAFE has been recognized as the main provider of information in relation to drug use and user profile within the Paris area. The steady increase in data available to the communities allows the different partners to find answers. SAFE is now regularly asked to provide data related to syringe distribution in order to answer critics. For example, the day and night monitoring of syringe exchange in various districts has clearly demonstrated the absence of activity at night time as well as a need to increase SDM capacities in some areas. Moreover, the 3.76 syringe collected per IDU disprove the syringe traffic flagged by local police authorities. But most of all, the high exchange rate and recycling of used syringes demonstrate the carefulness of the IDUs for health and safety issues!

**Table 2:** Data request

**Partners**	**Frequency**	**Data type**
National initiatives		
OFDT – TREND	3 times per year	Statistics
		Product use information
		User profiles
Coordination toxicomanies	Monthly	Statistics
		User profiles
Administration		
Health Regional Agency	5 times a year	Statistics
Paris City Council	5 times a year	Data on user profile evolution and product, biological data, data about cleanliness
Prefectures	Occasionally	
Organizations	4 times a year	Statistics
		Data on user profile evolution and product for specific areas

*OFDT* Observatoire Francais des Drogues et Toxicomanies (French Observatory on Drugs and Drug Addictions).

Also, this large dataset and its related possibilities have opened way to collaboration with several research groups, and new initiatives are being tested. For example, SAFE is using the SDM scheme and its knowledge of user profile (numbers, time between users, etc.) to analyze and define products found within the syringes with the Public Health Laboratory at University Paris XII - Châtenay-Malabry (unpublished). Similarly, an ongoing study is being run with the Virology Laboratory CERVI of Pitié-Salpêtrière Hospital, AP-HP on virus content within specific syringes collected at well-defined spots.

## Discussion

The surveys of SEPs are usually indirect [8]. They are evaluated via large virus infection surveys that cannot distinguish between SEP users and non-users. Another approach is the use of questionnaire that requires not only a trained interviewer but also willing users. This introduces a limitation as such. We took advantage of a large and well-defined SDM to test a different approach. We combined regular monitoring activities with counting devices to analyze the characteristics of such a SEP over a 10-year period.

Harm-reduction strategies are not constant over time, and the Parisian SEP is a clear illustration of the changes in activities and strategies of harm-reduction policy in the Paris area. However, the SDM-based SEP was maintained by the fusion of two related projects. This has allowed a follow-up of this strategy and an improvement of the SEP by promoting the coupled distributor/exchanger, relocating machines of low impact and replacing obsolete or poor-quality machines to improve the scheme. The increase in distribution as well as the recycling above the expected threshold is a positive result, but does not mean that the SAFE SEP is fully optimal. The variation per site is tremendous and is not fully understood. The introduction of counting device seems to provide a way to further understand the SEP and to hopefully provide optimal solutions.

The initial point of this study was to understand the SAFE SEP and to provide an objective view of its results and limitations to answer local concerns. Local authorities are regularly arguing against this SEP. However, most of their concerns could not be monitored previously and were based on observations or urban legends (the SEP is used by a limited amount of users, the SEP promotes syringe disposal in public areas, etc.). The mean kit uptake by users is 1.88, and the mean time between two collections that indicate two different users is above 10 min. This clearly indicates that IDUs in the Paris area only take a limited amount of syringes. We could conclude that this is an indication of limited shared syringes based on the limited amount of syringes taken; however, our analysis cannot be stretched this far, and further investigation using spectrometry and DNA-based methods will be needed to further characterize recycled syringes in correlation with collection. This could also be used to monitor addiction type per site. Our results proved the extended use of the SEP by IDU in Paris and answer objectively the local authorities' concerns.

Furthermore, we observed a recycling activity of the SEP which is above the expected threshold. And this effect increases over time. We already observed in a previous study a direct improvement of the cleanliness of the SDM SEP environment when distributors are coupled with exchangers [11]. It seems obvious that IDU knows about safety and disposal and consciously recycled their used syringes even without counterparts (coins). This recycling ability is higher than diabetes patients [12] or HVC-treated patients [13]. This result indicates a clear understanding of the SEP by its target population and a positive outcome that needs to be further investigated in terms of syringe exchange in between IDUs [14].

The annual variations of syringe distribution are limited and show an almost constant distribution activity. Only three slight variations can be observed. December and January, the coldest months of the year, show the lowest activity level, and we hypothesized a correlation between the slight drop of activity and the weather that may block machines (frozen drawers, etc.) and the effect on IDUs (susceptibility to cold when in need). This is partially confirmed by the limited number of IDUs met by the street team of SAFE (Table 3). The highest activity level is in April/May and July/August. The former increase in activity appears in a period with high number of public holidays (1st, 8th, 22nd, etc.) that limits access to other non-SDM SEPs and pharmacies. Also, the higher number of music festivals especially the electronic and techno music scenes are well known to attract IDUS, and so planning of drug consumption outside urban areas could lead to a tendency to stock kits prior to the event. Secondly, the July/August peak could be a side effect of the holiday seasons and the reduction of non-SDM-operated SEPs and pharmacies.

**Table 3:** User interaction with the SAFE team over the year 2009

**Month**	**Number of users**
January	76
February	78
March	85
April	101
May	*136*
June	103
July	*137*
August	93
September	111
October	150
November	113
December	98
Total	1,281

Highest activity is highlighted in italics.

Interestingly, the SAFE network data are slightly different from other national drug initiatives or local associations, which are probably in relation to the fact that the SDM set-up is a non-discriminatory system that attracts users outside the normal framework. We believe this is a more representative vision of injection practice. Moreover, the SDM set-up generates very large datasets within the 1,000-count range and over and so significantly higher than questionnaire-based studies. This provides better statistics and larger cohort study with limited bias.

The use of counting devices to monitor a SEP is the first time in the world; we are aware this has been done. This objective and innovative approach gave clear results concerning distribution rate and also distribution time window. Moreover, it illustrates our strategy. We have already observed an increase of supply disruptions in several sites, and public concerns pointed out an extensive use of the SDM during the night. However, we observed by monitoring heavily used sites (Gare du Nord and Barbes) a limited time window of distribution that ends up before night fall (19 h and 19 h 30 min, respectively). This disproves criticisms by local authorities that SDMs are heavily used at night and generate noise around machines, leading to neighbourhood complaints. But it can be seen as use of the SDMs by a population with a regular daily activity (workers, civil servants, etc.) that plan their syringe collection as part of their daily routine before leaving for the suburban areas of Paris. But it may point out towards a failure of the SAFE SEP to perform 24 h a day and 7 days a week. Already, SAFE has implemented a number of corrective measures:

Installation of dispensing machines of greater capacity in the Gare du Nord and Gare de l'Est. This has significantly improved the issue of supply disruptions (514 in 2007 against 19 in 2009), but continuous monitoring is currently ongoing to define the new time window of distribution and the efficiency of a higher distribution capacity

Gradual replacement of aging or obsolete machines, which has reduced the number of failures (570 in 2007 against 420 in 2009) and the number of technical interventions (1,202 in 2007 against 916 in 2009).

However, the disruption difficulties are not resolved because the disruptions now occur at scattered sites and irregularly, making the implementation of corrective actions difficult. Our approach is driven by the need to understand syringe distribution and recycling protocols to design the best strategy, and monitoring is an essential step to provide the highest standard of syringe accessibility. Several points are essential: shortest distance of an IDU to a machine, free products and multiplication of coin collection point (from 20 to 500), and the numbers we obtain seem to validate our strategic choices. It is important to use SDMs in our case as we can complement existing SEPs even during holidays and at nights and offer an alternative to IDUs including the non-French speaking group.

Our study took advantage of a well-developed SDM-based SEP to dissect the syringe/exchange distribution processes and to objectively analyze its efficiency. The use of simple counting devices allowed us not only to provide data to the SEP operator (SAFE) to better design their system but also to understand its limits (limited night distribution/machine capacity, etc.) and answer public concern (limited kit per IDU and higher recycling activity). Overall, this study demonstrates the need to monitor SEP activities to study harm-reduction strategies, answer public and politic concerns, and to develop new protocols.

## Conclusions

This report represents an unprecedented long-term analysis of a SDM SEP. Such a long and precise survey is a source of information to answer public and political concerns. It is also a valuable source of information for SEP curators in order to optimize their activities and better meet the needs of their users. Finally, it demonstrates the power of simpler and cheaper approaches to extract useful information from everyday use of machines and provide quantitative data to management, local authorities and police forces. We currently are developing our initial approach in combination with chemical and biological analyses [15] to further dissect the Parisian IDU population in regard to their consumption, needle sharing behaviour and infectious status down to the local scale.

## Abbreviations

HIV: Human immunodeficiency virus; HCV: Hepatitis C virus; IDU: Injection drug user; SEPs: Syringe Exchange Programs; SDM: Syringe-dispensing machine.

## Competing interests

The authors declare that they have no competing interests.

## Authors' contributions

CD and EGR designed the study. CD supervised the data collection. CD and EGR planned the analysis of the manuscript. EGR conducted the statistical analysis and wrote the first draft of the manuscript. Both authors contributed to the final version of the manuscript. Both authors read and approved the final manuscript.
